# Fluorescence Study of Riboflavin Interactions with Graphene Dispersed in Bioactive Tannic Acid

**DOI:** 10.3390/ijms22105270

**Published:** 2021-05-17

**Authors:** María Paz San Andrés, Marina Baños-Cabrera, Lucía Gutiérrez-Fernández, Ana María Díez-Pascual, Soledad Vera-López

**Affiliations:** 1 Universidad de Alcalá, Facultad de Ciencias, Departamento de Química Analítica, Química Física e Ingeniería Química, Ctra. Madrid-Barcelona Km. 33.6, 28805 Alcalá de Henares, Madrid, España (Spain); marineta.bc@gmail.com (M.B.-C.); lucia.gutierrezf@edu.uah.es (L.G.-F.); am.diez@uah.es (A.M.D.-P.); soledad.vera@uah.es (S.V.-L.); 2 Universidad de Alcalá, Instituto de Investigación Química Andrés M. del Río (IQAR), Ctra. Madrid-Barcelona Km. 33.6, 28805 Alcalá de Henares, Madrid, España (Spain)

**Keywords:** tannic acid, graphene, fluorescence, quenching, riboflavin

## Abstract

The potential of tannic acid (TA) as a dispersing agent for graphene (G) in aqueous solutions and its interaction with riboflavin have been studied under different experimental conditions. TA induces quenching of riboflavin fluorescence, and the effect is stronger with increasing TA concentration, due to π-π interactions through the aromatic rings, and hydrogen bonding interactions between the hydroxyl moieties of both compounds. The influence of TA concentration, the pH, and the G/TA weight ratio on the quenching magnitude, have been studied. At a pH of 4.1, G dispersed in TA hardly influences the riboflavin fluorescence, while at a pH of 7.1, the nanomaterial interacts with riboflavin, causing an additional quenching to that produced by TA. When TA concentration is kept constant, quenching of G on riboflavin fluorescence depends on both the G/TA weight ratio and the TA concentration. The fluorescence attenuation is stronger for dispersions with the lowest G/TA ratios, since TA is the main contributor to the quenching effect. Data obey the Stern–Volmer relationship up to TA 2.0 g L^−1^ and G 20 mg L^−1^. Results demonstrate that TA is an effective dispersant for graphene-based nanomaterials in liquid medium and a green alternative to conventional surfactants and synthetic polymers for the determination of biomolecules.

## 1. Introduction

Graphene (G) is a carbon nanomaterial that comprises a two-dimensional honeycomb lattice of sp^2^ hybridized carbon atoms with one-atom thickness, a high electron density, and a large surface area [1]. It has excellent electrical, optical, and thermal properties, combined with high mechanical resistance, transparency, low density and flexibility [2]. The G structure with delocalized π bonds accounts for its high electrical and thermal conductivity [3]. Its properties make it an ideal material for a wide range of applications, ranging from structural nanocomposites [4], to electronics, optics, sensors, and biodevices.

G can interact covalently and non-covalently with other molecules or polymers and is able to incorporate them on both sides of its sheets. Covalent interactions occur via formation of a chemical bond, and two main approaches can be discerned: grafting-from and grafting-to [5]. In the former, G is used as a growing platform of the molecules or polymer chains, whereas the later consists of the direct coupling of G or pre-functionalized G with the polymer chains, which should incorporate reactive functional groups. Nonetheless, these strategies can alter the conjugated π-system of G and bring together structural defects that led to worse performance. On the other hand, the non-covalent approach is based on the physical adsorption and/or wrapping of molecules or polymers via weak interactions such as H-bonding, hydrophobic (van der Waals) π-π, H-π, cation-π, and anion-π, which preserve the intrinsic electronic properties of this nanomaterial. G has high surface adsorption capacity, and is able to retain molecules with different structures, especially those with aromatic rings [6]. Further, it is hydrophobic and has a strong tendency to agglomerate, so it must be dispersed in liquids (such as organic solvents or water) frequently with the aid of ultrasounds and/or dispersing agents [7,8]. Different dispersants have been used to improve their solubility and other properties. Good aqueous dispersions of G sheets have also been achieved using a derivative of pyrene, 1-pyrenebutyrate [9]. Surfactants are amongst the most widely used dispersing agents for G in aqueous media. Sodium cholate with mild sonication for a long time disperses G up to concentrations of 0.3 mg mL^−1^ [10]. Cationic, anionic, and nonionic surfactants have been used [11], and while ionic surfactants adsorbed on G provide electrostatic repulsion between sheets that prevent their aggregation, nonionic ones lead to stabilization by steric interactions [12]. Nonionic surfactants (such as Polyoxyethylene-100-stearyl ether (Brij 700) or Poly(ethylene glycol)-block-poly(propylene glycol)-block-poly(ethylene glycol) (Pluronic P-123) can exfoliate and disperse G in water at high concentrations, while ionic surfactants such as sodium taurodeoxycholate (TDOC) or sodium dodecyl sulfate (SDS) are less effective [13]. In our research group, the ability of surfactants of different natures (cationic hexadecyltrimethylammonium bromide (CTAB), zwitterionic laurylsulfobetaine (LSB), and nonionic polyoxiethylen-23-lauryl ether (Brij L23) to disperse G in an aqueous medium has been thoroughly investigated. Further, the interaction of G with α-tocopherol [14], with the anionic surfactant SDS, or with pyrene [15], as well as their interaction with vitamins, such as riboflavin [16] and pyridoxine [17], have been investigated in detail. The ability of surfactants to disperse G depends on a number of parameters, including their charge, concentration, and length of the hydrocarbon chain. Polymers, such as polyethylenglicol (PEG) and copolymers such as polyethylene glycol-polypropylene glycol-polyethylene glycol (poloxamer P-407), have also been studied to disperse G and graphene oxide (GO) [18,19].

**Figure 1:**
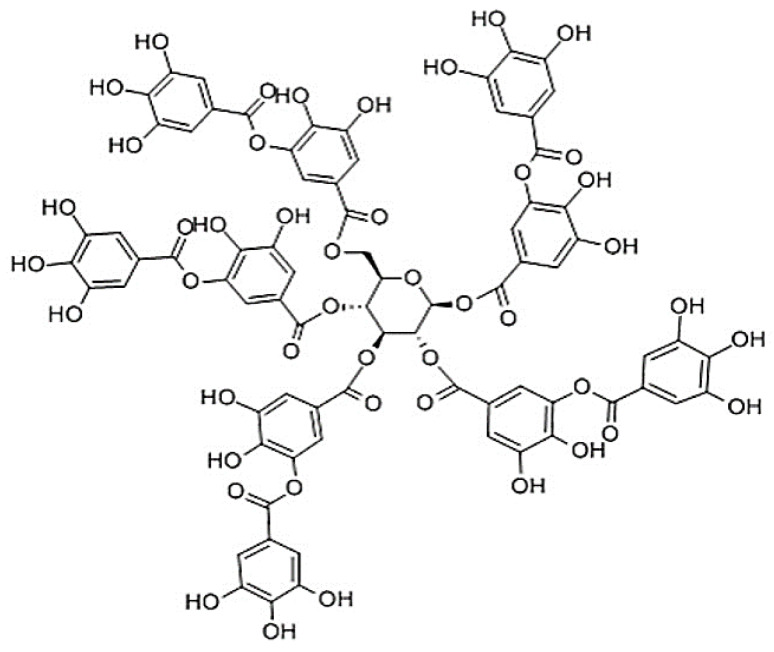
Chemical structure of tannic acid.

Tannic acid (TA) is a water-soluble polyphenolic compound that belongs to the group of hydrolysable tannins, found naturally in fruits, seeds, plants, and tree bark. It is present in foods such as wine, coffee, tea, and beer. It has antibacterial, antienzymatic, and astringent properties, although it inhibits the absorption of iron in the body by complex formation. It is also used to treat skin ulcers, wounds, and toothaches. Further, it is employed as a food additive (E-181), and it is a clarifying agent and flavor enhancer [20]. Its chemical structure (Figure 1) consists of a central glucose molecule, which hydroxyl groups are linked by ester bonds with gallic acid moieties. One molecule contains 8 to 10 moles of gallic acid per mole of glucose [21]. In the literature, different pK_a_ values for TA have been reported, most recently, 6.3, 7.4, and 8.6 [22]. In a previous study [23], TA was used as an additive to modify the structure of a graphene derivative (GO) for the development of GO/carbonized paper/TA ternary composites in the solid state to be used as flexible electrodes in energy storage applications. TA molecules adsorbed onto the GO via π-π stacking interactions and increased the interlayer spacing between GO sheets, hence the contact area with the carbonized paper. On the other hand, due to its structure and biocompatibility, TA is a good candidate to replace synthetic surfactants in order to obtain low-cost, environmentally friendly G dispersions in water. TA has been used to obtain G by exfoliating graphite with ultrasound, and the influence of TA concentration, pH, and sonication time have been studied, among other parameters [24]. Other polyphenols such as resveratrol [25], and even raw tea [26] or coffee [27], have also been used for the dispersion of G and its derivatives.

Riboflavin, known as vitamin B_2_, is a molecule of biological interest, a water-soluble vitamin excreted in the urine and an essential nutrient, since its daily consumption is necessary. It is present in the body principally as a basic constituent of the coenzymes flavin adenine dinucleotide (FAD) and flavin mononucleotide (FMN), and it participates in cell development and human growth [28]. Further, it contributes to the metabolism of other vitamins, carbohydrates and proteins, it participates in multiple metabolic reactions, such as the formation of red blood cells, functioning of the nervous system, and DNA synthesis [29], it is essential for energy production, and acts as an antioxidant. Riboflavin scarcity can induce hematologic, cardiovascular, and gastrointestinal diseases and has been identified as a risk factor for cancer [30]. Henceforth, considering the great nutritional significance of this water-soluble vitamin, it has been taken as model in this study.

Riboflavin has native fluorescence, which has been studied in different media. Some amino acids, such as cysteine and methionine, produce the attenuation of the fluorescence of riboflavin, known as “quenching” [31], which can be produced by energy transfer (dynamic quenching) or by formation of a ground state complex (static quenching) [32]. In both cases, a linear relationship can be obtained between the ratio of the fluorescence intensity in the absence (F_0_) and in the presence of the quencher molecule (F) and the quencher concentration ([Q]). The intercept of the plot should be equal to one, and the slope is the Stern–Volmer constant (K_SV_) in the dynamic quenching Equation (1), being K_SV_ = τ K_q_ (τ = fluorescence lifetime, K_q_ = bimolecular quenching constant) while in the static quenching K_SV_ is replaced by K_S_ Equation (2), the constant of formation of the complex:

F_0_/F = 1 + k_q_ τ_0_ [Q] = 1 + K_SV_ [Q] (1)

F_0_/F = 1 + K_S_ [Q] (2)

Steady-state fluorescence measurements do not allow distinguishing between the two types of quenching, hence lifetime measurements are necessary. Nevertheless, the quenching constants (either dynamic (K_SV_) or static (K_S_)), can be calculated from the plot of F_0_/F versus [Q], which provides information about the interactions between the analyte and the quencher molecule.

The main aim of this work is to investigate the possibilities of tannic acid as a dispersant agent for G in water, a biocompatible medium. Fluorescent measurements have been carried out to study the interaction between G dispersed in tannic acid and a fluorescent molecule of high biological interest, such as riboflavin. Thus, the interaction of this vitamin with tannic acid or G/tannic acid system has been evaluated and the results have been compared with those previously obtained for G dispersed in synthetic surfactants and polymers, in order to assess the effectiveness of tannic acid as dispersing agent.

## 2. Results and Discussion

### 2.1. Riboflavin Fluorescence in Water and in TA Aqueous Solutions without G

**Figure 2:**
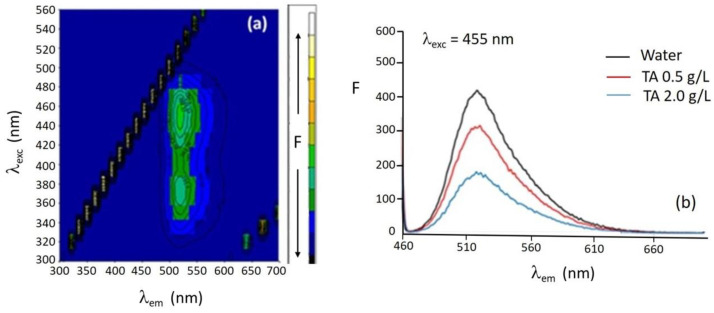
3D fluorescence spectrum of riboflavin 0.6 mg L^−1^ in water (**a**) and emission spectra at λ_exc_ 455 nm in water and tannic acid 0.5 y 2.0 g L^−1^ (**b**).

The fluorescence three-dimensional spectrum of riboflavin 0.6 mg L^−1^ in water is shown in Figure 2a. Two fluorescence intensity maxima are observed at excitation wavelengths of 380 nm and 455 nm. The emission maxima are found at 520 nm for the two excitation wavelengths. The excitation and emission maximum wavelengths hardly shift upon changing the medium from water to TA aqueous solutions at pH 4.1. Figure 2b shows the emission spectra at l_exc_ = 455 nm of riboflavin in water and in the presence of 0.5 and 2.0 g L^−1^ TA aqueous solutions. A decrease in the fluorescence intensity of riboflavin is observed with increasing TA concentration due to the interaction between both compounds. A fluorescence quenching phenomenon occurs owing to the interaction between TA and riboflavin that can take place via π-π stacking through the aromatic rings, via hydrogen bonding between the hydroxyl moieties of both molecules, or even via hydrophobic or electrostatic forces. TA presents the ability of multiple hydrogen bonding and crosslinking due to its numerous hydroxyl groups, and the pH and temperature dependence of its interactions with biomolecules [33] or polymers [34] have been reported.

The fluorescence spectra obtained in water, as well as in 0.5 and 2.0 g L^−1^ TA solutions, are similar at pHs of 4.1 and 7.1. A clear decrease in the intensity is observed with increasing TA concentration, which causes fluorescence quenching of riboflavin. The ratio between the fluorescence intensity of riboflavin in water and in the presence of TA solutions at the maximum emission wavelength (520 nm) for the excitation wavelength of 455 nm are calculated, and the results are shown in Table 1.

The decrease in fluorescence intensity in the presence of TA is significantly stronger for 2.0 g L^−1^ than for 0.5 g L^−1^ at both pHs. Therefore, it can be concluded that the interaction between both molecules occurs at both pHs, either by energy transfer from riboflavin in the excited state to TA (dynamic quenching), or by formation of a complex in the ground state between both molecules (static quenching). As indicated above, both molecules can interact mainly via π-π and H-bonding interactions. The fact that there is hardly any difference between the fluorescence quenching found at pH 4.1 and 7.1 suggests that π-π interactions predominate. Nonetheless, hydrogen bonds should also play a key role in the interaction between both molecules. Our results are in agreement with former works that studied the interactions between molecules with hydroxyl moieties and polyphenols; these weaken with increasing temperature, but depend only slightly on the solution pH [34,35].

**Figure 3:**
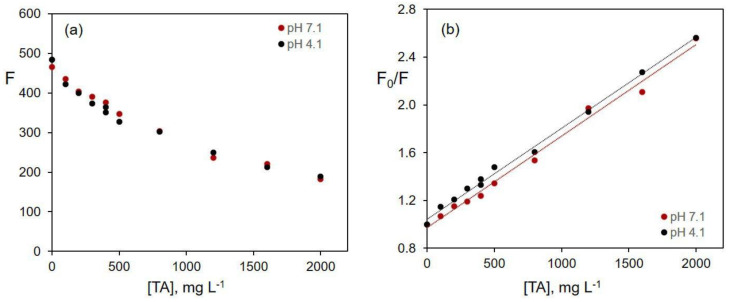
Riboflavin (0.6 mg L^−1^) fluorescence intensity versus TA concentration (**a**) and Stern–Volmer plot (**b**). Each data point is the average of at least 2 independent measurements.

To assess the influence of TA concentration on the quenching of riboflavin fluorescence intensity, solutions with TA concentrations ranging from 0 to 2.0 g L^−1^ were prepared, and the Stern–Volmer equation was plotted. Figure 3a shows the decrease in fluorescence intensity as a function of TA concentration and Figure 3b shows the F_0_/F ratio for increasing TA concentrations, where F is the intensity of riboflavin in TA and F_0_ is the fluorescence intensity of riboflavin in water in the absence of TA at pHs of 4.1 and 7.1.

As can be observed in Figure 3b, the fluorescence quenching produced by TA on riboflavin follows the Stern–Volmer equation and the F_0_/F ratio is linear up to at least 2.0 g L^−1^ of TA. The behavior is similar for both pHs, indicating that the dissociation of some TA hydroxyls does not influence the interaction between the two molecules.

The calculated values for the intercept, the slope with its standard deviations (SD), and the correlation coefficient (r), as well as the values of the quenching constants (K), either dynamic or static (K_SV_ or K_S_) for both pHs, are collected in Table 2.

The quenching constants expressed considering the molar concentrations of TA are 1404 M^−1^ and 1348 M^−1^ for pHs of 4.1 and 7.1, respectively.

### 2.2. Riboflavin Fluorescence in the Presence of Graphene Dispersions in Tannic Acid 2.0 g L^−1^

**Figure 4:**
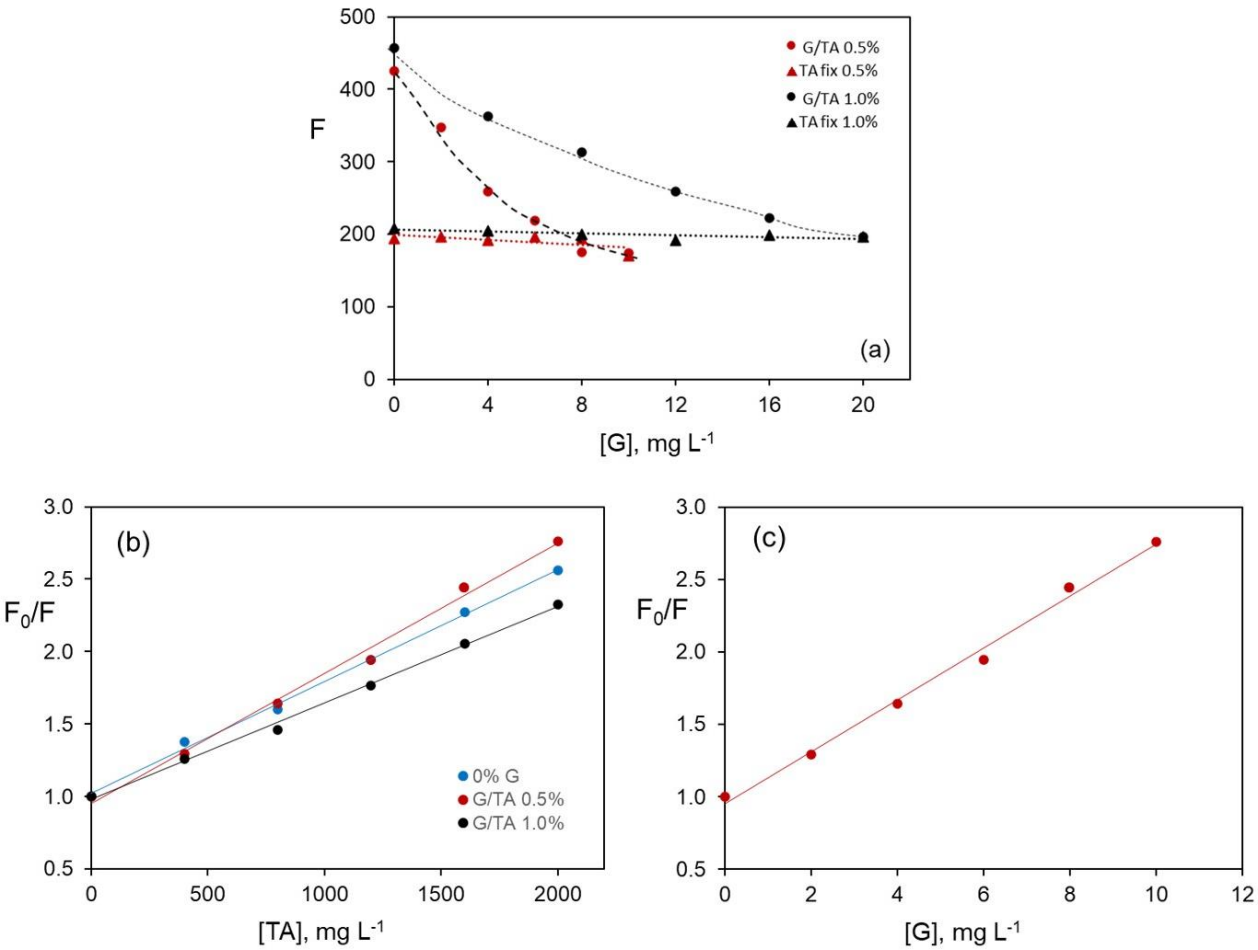
Change in F as a function of G concentration (**a**); change in F_0_/F versus TA concentration (**b**); F_0_/F versus G concentration (**c**) for G dispersions in TA 2.0 g L^−1^, pH of 4.1 ([Riboflavin] = 0.6 mg L^−1^; λ_ex_ = 455; λ_em_ = 520 nm).

The dispersions of G in TA were prepared at pH 4.1, which is the pH of the aqueous solution of 2.0 g L^−1^ TA. Appropriate amounts of G were added to obtain G/TA mass ratios of 1.0% and 0.5%. The dispersions were prepared by keeping the G/TA mass ratio constant, which implies that the concentrations of G and TA vary when making the corresponding dilutions with water, and with a variable mass ratio G/TA, using TA 2.0 g L^−1^ as solvent. All the obtained dispersions were found to be stable, that is, they did not settle even after a few days (see photographs in Figure S1). Figure 4 shows the variation of the fluorescence intensity of riboflavin, for the two G percentages tested, versus G concentration (Figure 4a), the change in the F_0_/F ratio versus TA concentration (Figure 4b), and as a function of G concentration (Figure 4c). In all cases, a distinction is made between a G/TA mass ratio constant or variable.

From the results in Figure 4a, for a variable G/TA mass ratio that is fixed TA concentration (data marked with black and red triangles), the presence of G does not influence the decrease in riboflavin fluorescence. For both data series, the fluorescence values are almost the same to that obtained in the presence of an aqueous solution of TA 2.0 g L^−1^.

However, when the G/TA mass ratio is constant (data marked with red and black circles), two sets of results are observed. For a G concentration of 1.0 wt%, the fluorescence values of riboflavin are the same as those obtained in the absence of G (Figure 3a), indicating again that G does not produce noticeable additional quenching to that found in TA solutions. A more pronounced quenching is observed for a G concentration of 0.5 wt%, probably due to the sum of contributions of G and TA. Assuming that for a constant G concentration of 1.0 wt% the predominant quenching is due to TA, Figure 4b shows the Stern–Volmer graphs, where slight modifications can be observed in the values of the quenching constants with the percentage of G. In any case, this modification is insignificant, and it can be considered that the constants are almost the same.

In the presence of a G concentration of 0.5 wt% there seems to be an additional quenching to that produced by TA, as shown in Figure 4a. If so, Stern–Volmer can be applied assuming that G is responsible for the process. Figure 4c shows the Stern–Volmer graph, the data are adjusted with a correlation coefficient of 0.997 and yield a quenching constant value of 0.0009 L mg^−1^ in G 0.5 wt%, about 29% higher than that obtained if the TA were the only quencher (0.0007 L mg^−1^ in the absence of G).

From the results obtained, the role of G in the quenching process is not clear. This may be due to an inadequete dispersion of G in the TA solutions, to the existence of notable differences in how TA interacts with G that causes poor accessibility of riboflavin to the dispersed G sheets, to the influence of pH, the TA concentration, or the G weight percentage. To try to elucidate these questions, a series of experiments introducing various variables by performing G dispersion in aqueous TA solutions are carried out, as detailed below.

The fluorescence of riboflavin decreases compared to that obtained in water by a factor F/F_W_ = 0.43 in the presence of TA for the two G percentages studied, but remains constant as the G concentration is increased, with F/F_0_ = 1.0. However, for dispersions obtained via dilution with TA of the same concentration as the dispersion, no decrease in the fluorescence of riboflavin (0.6 mg L^−1^) is observed, which should be related to the presence of G. This fact is corroborated by comparing with the results obtained for dispersions prepared by diluting with water. In this series, the fluorescence intensity decreases with increasing G and TA concentration up to the same value obtained for the G dispersion in 2.0 g L^−1^ TA.

**Scheme 1:**
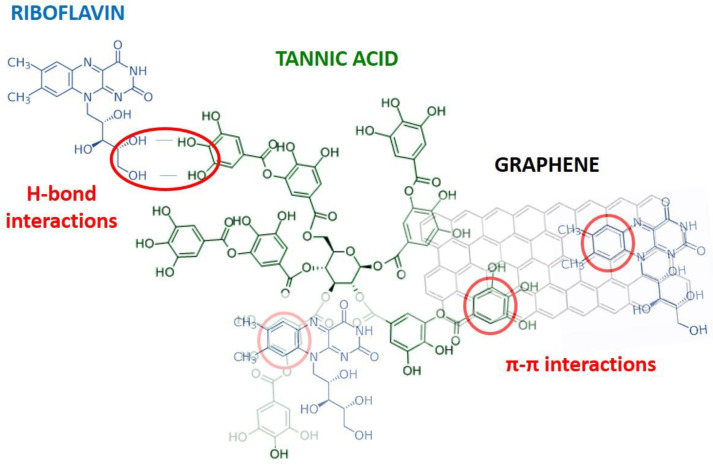
Illustration of the interactions among G, TA, and riboflavin.

Results suggest that at pH of 4.1, G dispersed in TA scarcely interacts with riboflavin, hence no change in its fluorescence is observed, either because G is not properly dispersed, or because the amount dispersed in this medium is very low. The interaction of riboflavin with TA is much stronger than that of riboflavin with G dispersed under these conditions. Therefore, it can be concluded that the quenching of TA on riboflavin occurs through the hydroxyl moieties of ribose and not via the aromatic rings, since G has a large number of aromatic rings all throughout its structure. A chemical model illustrating the potential interactions among G, TA, and riboflavin is shown in Scheme 1.

It can be inferred that the quenching phenomenon of riboflavin fluorescence in the G dispersions is due to the presence of TA, hence the quenching constants obtained decrease with increasing G concentration. Thus, as the G/TA weight ratio increases from 0.5% to 1.0%, the higher the G concentration in the dispersion, the smaller the slopes of the Stern–Volmer plots, that is, the lower the quenching constants. Therefore, it is found that the quenching magnitude is inversely proportional to the G concentration, while it is directly proportional to the TA concentration in each solution. This is due to the fact that the lower the concentration of G, the higher the TA concentration, and TA is a more efficient quencher than G.

The values of the intercepts, slopes along with their standard deviations, correlation coefficients, and quenching constants obtained from the Stern–Volmer plot for G dispersions (0.5 wt% and 1.0 wt%) in TA at a concentration of 2.0 g L^−1^ and at pH of 4.1, are collected in Table 3. Values at the top and bottom of the table correspond to dispersions with a constant TA concentration and a constant G/TA weight ratio, respectively.

It can be observed that the values of the quenching constants do not change for the series with a constant TA concentration, regardless of the percentage of G in the dispersion. However, when the G/TA weight ratio is maintained, the quenching constants decrease since G concentration is double in the dispersion with 0.5%, compared to that with 1.0% for the same TA concentration (2.0 g L^−1^). Therefore, it is confirmed that the quenching observed is caused by the TA, since the quenching constants decrease when increasing the amount of G in the dispersion (i.e., K decreases by a factor near 2 as G concentration is doubled). To make it clearer, the parameters obtained upon increasing G concentration are highlighted in blue, since they do not correspond to the quenching produced by G on riboflavin, but to the quenching induced by TA.

### 2.3. Study on the Improvement of G Dispersions in Aqueous Solutions of TA

#### 2.3.1. Influence of the Sonication Time on the Preparation of 0.5 wt% G Dispersion in TA 2.0 g L^−1^ at pH 4.1

The optimization of the parameters influencing the preparation of the G dispersions is crucial to attain dispersions with optimal properties. Among them, the time of application of the ultrasonic probe to the mixture can be crucial. With the aim to elucidate whether the increase in the sonication time, during the preparation stage, can improve the state of dispersion of G in TA at pH 4.1, as has been previously reported for the exfoliation of graphite in TA [23], a similar experimental procedure to that previously described was used, albeit increasing the time the probe was applied to the G/TA mixture. Thus, the sonication time was increased from 5 to 15 min, and the fluorescence intensity obtained for both cases is shown in Figure S2.

No significant change in riboflavin fluorescence is observed with increasing probe sonication time; therefore, henceforth all dispersions have been prepared with a probe time of 5 min.

#### 2.3.2. Influence of Solution pH on the Fluorescence of Riboflavin in the Presence of G Dispersions in TA 2.0 g L^−1^

With the aim to improve the dispersion of G in TA solutions, the solution pH was increased to 7.1, at which, part of the hydroxyl groups of TA are dissociated. Dispersions are prepared following the same protocol. Upon preparation of the dispersions, two series of measurements are carried out, similarly to those made at pH 4.1, keeping the mass ratio G/TA constant, which implies that the concentrations of G and TA vary when making the corresponding dilutions with water, and with a variable mass ratio G/TA, using TA 2.0 g L^−1^ as solvent.

**Figure 5:**
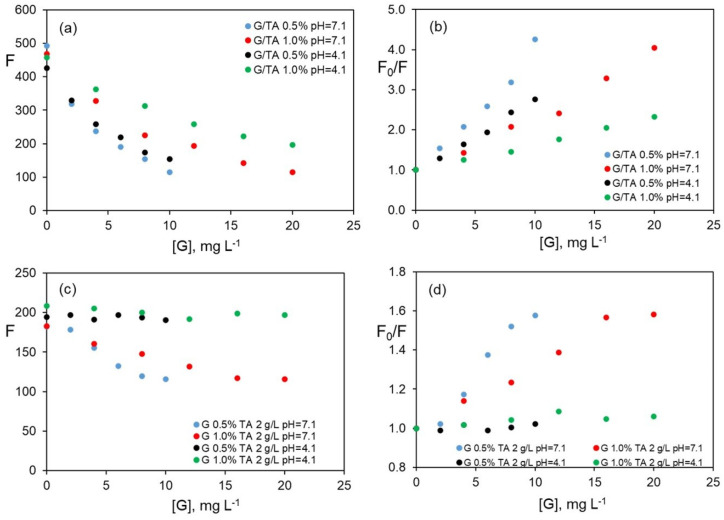
Variation of F (**a**) and F_0_/F (**b**) versus [G] at a constant G/AT ratio and F (**c**) and F_0_/F (**d**) versus [G] at constant [TA] for G dispersions in TA 2 g L^−1^.

Figure 5 shows the comparison of the results previously obtained at pH 4.1 and those obtained at pH 7.1.

The change in the fluorescence intensity of riboflavin, λ_exc_/λ_em_ = 455/520 nm, versus G concentration for dispersions prepared in TA 2.0 g L^−1^, weight ratio G/TA constant (G 0.5% and 1.0% wt%) at the two working pH, is displayed in Figure 5a.

As can be observed, a stronger decrease in the fluorescence of riboflavin at pH 7.1, for the constant G/TA mass ratio and a 1.0 wt% of G, is observed. However, when the percentage of G is 0.5, no significant differences for both pH values are observed.

Figure 5b shows the fit to the Stern–Volmer equation for both pH values. Contrary to the behaviour found at pH 4.1, the data do not fit the model at pH 7.1, with an intercept value different from unity.

Figure 5c shows the results obtained for the change in fluorescence when the G/TA mass ratio is variable and the TA concentration is constant, for pH values of 4.1 and 7.1. Figure 5d shows the fit to the Stern–Volmer equation. In this case, higher quenching also occurs at pH 7.1.

Although the results are not conclusive, it is possible to assume that at pH 7.1, the interaction of a more dissociated TA with G allows for dispersions more accessible to the riboflavin molecule. Quenching is a result of two types of interactions, through the hydroxyl moieties of ribose of TA and via the aromatic rings of G. Therefore, there is a double contribution to the quenching phenomenon of riboflavin fluorescence.

#### 2.3.3. Influence of TA Concentration in the G Dispersions on the Fluorescence of Riboflavin at pH 7.1

**Figure 6:**
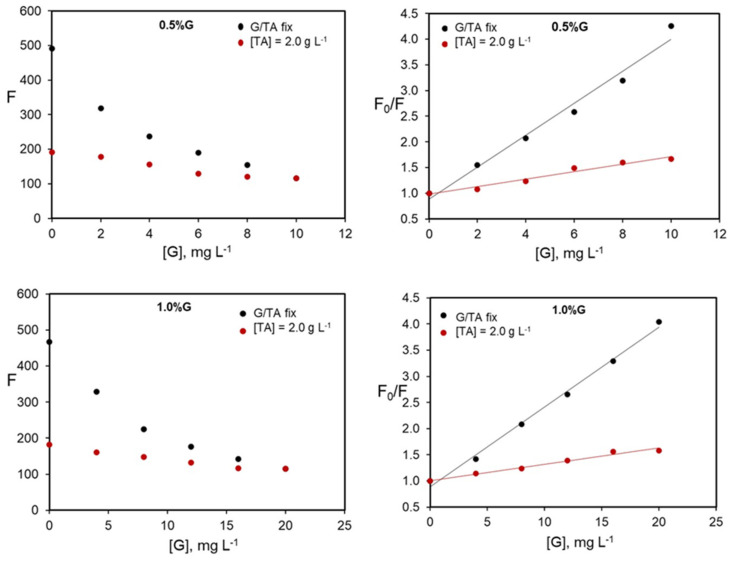
Change in the fluorescence intensity of riboflavin (0.6 mg L^−1^) and F_0_/F ratio as a function of G concentration for G dispersions 0.5 y 1.0 wt% in TA 2 g L^−1^ (λ_ex_/λ_em_ = 455/520 nm, pH = 7.1).

As can be observed in Figure 6, at pH 7.1 a fluorescence quenching phenomenon caused by the presence of G takes place, in addition to that caused by TA in the absence of the nanomaterial.

In solutions with a constant TA concentration (2.0 g L^−1^), the decrease in the fluorescence of riboflavin is the same for the two G percentages studied, although the G concentration for the dispersion with 1.0% (20 mg L^−1^) is twice that for 0.5% (10 mg L^−1^).

**Figure 7:**
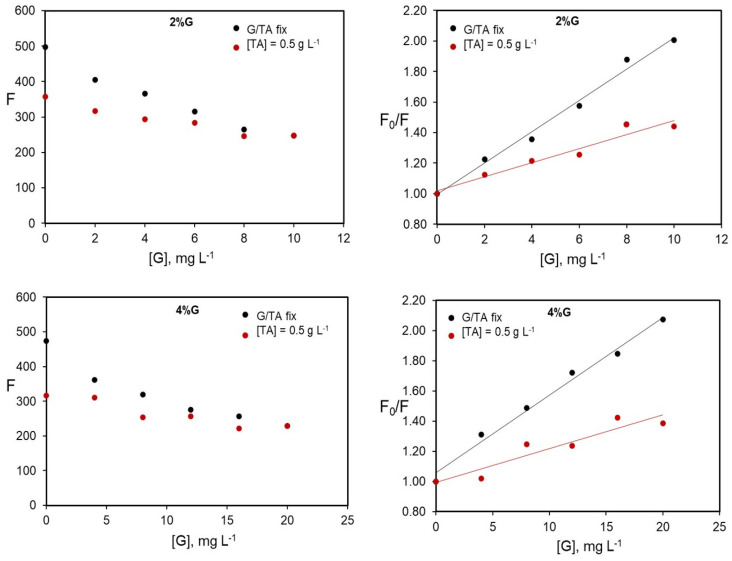
Change in the fluorescence intensity of riboflavin (0.6 mg L^−1^) and F_0_/F ratio as a function of G concentration for G dispersions 2.0 wt% and 4.0 wt% in TA 0.5 g L^−1^ (λ_ex_/λ_em_ = 455/520 nm; pH = 7.1).

In order to study the effect of TA concentration in the G/TA dispersions on the fluorescence of riboflavin, two sets of dispersions were prepared with a TA concentration of 0.5 g L^−1^ and G weight ratios of 2.0% and 4.0%. These percentages correspond to the same G concentrations added in TA dispersions 2.0 g L^−1^, for G weight ratios of 0.5 and 1.0% *w*/*w*, setting the pH at 7.1. The results are plotted in Figure 7. By comparing the change in the fluorescence at a variable and constant TA concentration, it is possible to determine whether the interaction of riboflavin with G depends on the concentration of G and tannic acid or only on the weight ratio between both compounds.

The comparison of Figure 6 and Figure 7 reveals that for the same G concentrations, dispersions in TA 0.5 g L^−1^ lead to a smaller decrease in riboflavin fluorescence than those prepared in TA 2.0 g L^−1^. This behaviour was expected, since TA is the main factor responsible for the decrease in riboflavin fluorescence intensity.

For the two G percentages studied, smaller slopes are found for a TA concentration of 0.5 g L^−1^ and, as mentioned previously, for a TA concentration of 0.5 g L^−1^, the slope found for dispersions with a G/TA weight ratio of 4.0% is approximately half that found for dispersions with a weight ratio of 2.0%.

The decrease in the intensity of riboflavin fluorescence is considerably stronger when TA concentration changes than when it remains constant. While TA interacts strongly with riboflavin, the interaction of G with the vitamin is much weaker. This behavior is the same for G dispersions in TA 0.5 and 2.0 g L^−1^. When TA concentration (0.5 g L^−1^) is constant, a straight line is obtained with a small slope, lower than that in TA 2.0 g L^−1^. In addition, for this TA concentration, data variability is greater, especially for the dispersion with a G/TA weight ratio of 4.0%, which was tested three times to check for reproducibility and in all cases the data lead to a poor linear fit.

**Figure 8:**
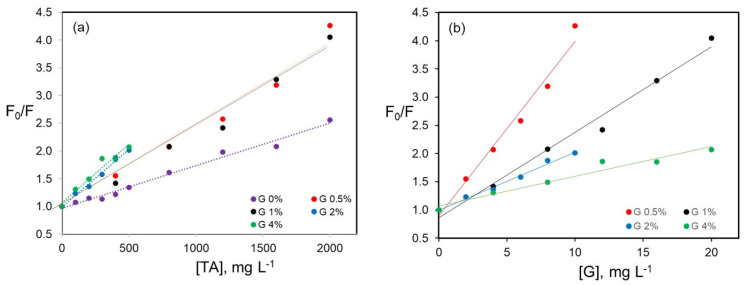
F_0_/F ratio as a function of TA concentration (**a**) and G concentration (**b**). [Riboflavin] = 0.6 mg L^−1^, λ_ex_/λ_em_ = 455/520 nm, pH = 7.1.

Figure 8 shows F_0_/F ratio (Stern–Volmer plot) as a function of G and TA concentration for G dispersions in TA 2.0 g L^−1^ and TA 0.5 g L^−1^ and the four percentages of G (0.5, 1.0, 2.0 and 4.0%) for a constant G/TA ratio. The values in the absence of G have also been included for comparison.

It can be observed from Figure 8a that the presence of G in the medium increases the quenching effect, and as the concentration of TA in the medium increases, this effect is less pronounced than that found for the lowest concentration, 0.5 g L^−1^. Further, for the same TA concentration, the F_0_/F ratio does not depend on the G percentage. On the other hand, when F_0_/F ratio is plotted against the G concentration, the slope decreases as the percentage of G in the dispersion increases, regardless of the TA concentration.

**Figure 9:**
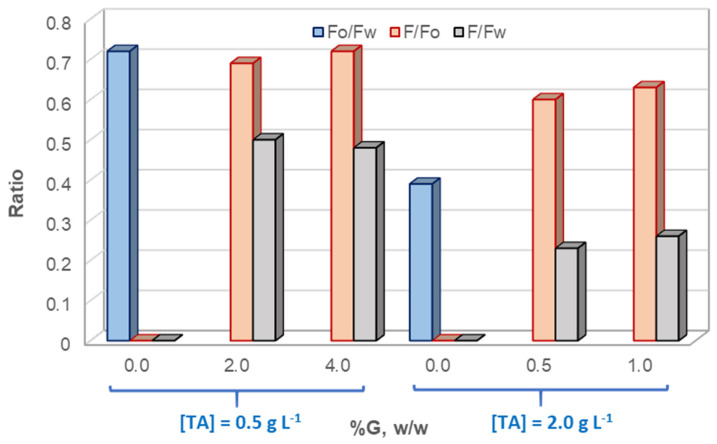
Plot of the fluorescence intensity ratios of riboflavin in the different media. F_W_: water; F_0_: Tannic Acid; F: G/TA dispersions.

The fluorescence intensity ratios (F_o_/F_w_, F/F_o_ and F/F_w_), obtained for the two TA concentrations and the different G weight percentages studied in this work, are compared in Figure 9.

It can be observed that the quenching effect caused by TA is directly proportional to its concentration (28% and 62% in TA 0.5 and 2.0 g L^−1^, respectively). An additional decrease in riboflavin fluorescence is found in the presence of G, and the effect is independent to the G percentage, while proportional to the TA concentration.

The same behaviour is found when the quenching efficiency in TA solutions is compared with that obtained for G dispersions in TA, regardless of the percentage of G. Thus, the quenching is weaker for dispersions in TA 0.5 g L^−1^ (efficiency of 50% and 52% for G percentages of 2.0% and 4.0%, respectively) than in 2.0 g L^−1^ (efficiency of 77% and 74% for the same G percentages). This suggests that the interaction with riboflavin is stronger in the dispersions with the highest TA concentration. Finally, for dispersions with a constant G/TA ratio, the fluorescence quenching (i.e., F/F_W_) is again independent to the G percentage in the dispersion with increasing TA concentration. In this case, the decrease in fluorescence intensity corresponds to a synergistic effect of both G and TA in the dispersion.

### 2.4. Quenching Constants of Riboflavin in the Presence of G Dispersions in TA for Variable TA Concentration

Figure 9 shows that the quenching effect induced by TA considerably increases in the presence of G, albeit does not depend on the G/TA weight ratio in the dispersion.

Table 4 lists the fluorescence quenching constants of riboflavin for G dispersions in TA 2.0 and 0.5 g L^−1^ for different G/TA weight ratios, for variable G and TA concentrations. The coefficients of the linear fits along with their standard deviations (SD) are also included. In all cases, points that deviated from the linearity were removed.

The parameters obtained upon increasing G concentration are again highlighted in blue, since these also include the influence of TA on the vitamin fluorescence intensity. The effect of both G and TA concentration has been simultaneously assessed using multiple regression for a 95% confidence level, and the regression coefficients obtained along with their confidence interval (CI), are displayed in Table 5.

The experimental values of F_0_/F as a function of the predicted values by the multiple linear regression are plotted in Figure S3, showing a correlation of 95%. As can be observed in Table 5, this equation allows for the calculation of the simultaneous effect of TA and G upon the fluorescence of riboflavin, evaluating the quenching contribution of each variable.

When F_0_/F values are plotted independently versus TA concentration, quenching constants of 0.020 and 0.014 L mg^−1^ are found for TA concentrations of 0.5 and 2.0 g L^−1^, respectively, irrespective the percentage of G in both cases. For G, K values of 0.27 and 0.15 L mg^−1^ are obtained for dispersions with 0.5% and 1.0% G in TA 2.0 g L^−1^, respectively. However, those with 2.0% and 4.0% G in TA 0.5 g L^−1^ show smaller K values, 0.10 and 0.05 L mg^−1^, respectively. It is difficult in this case to separate the effect of TA and G, since the concentrations of both compounds varies simultaneously. In the multiple regression analysis, the influence of both concentrations on the F_0_/F ratio is taken into account, and a linear relationship is obtained with an intercept that does not statistically differ from zero. Further, the coefficients are 0.05 for G, which is the lowest value of those obtained with the two concentrations of TA, and 0.0009 for TA, which is very close to that obtained for this compound in the absence of G. It can be concluded that the presence of G enhances the riboflavin-TA interaction, albeit it appears that TA is the main contributor to the quenching effect. This is consistent with the fact that the slope of F_0_/F versus G concentration is considerably higher than that versus TA.

### 2.5. Quenching Constants of Riboflavin in the Presence of G Dispersions in TA for a Constant TA Concentration

**Figure 10:**
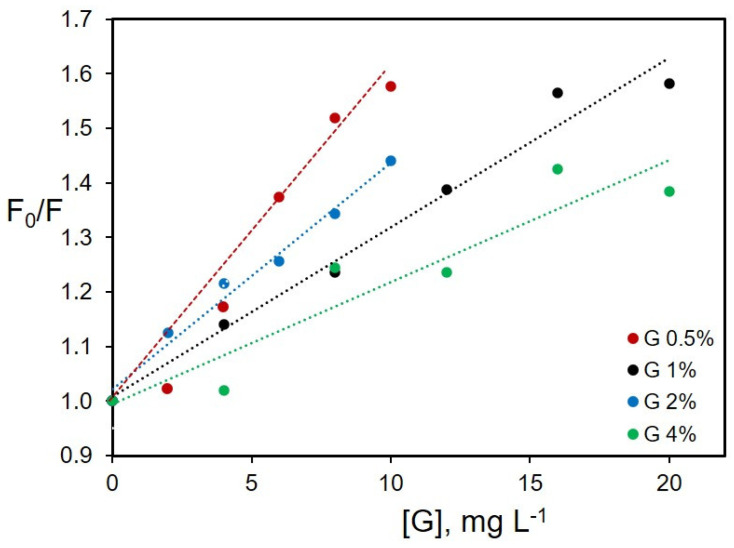
F_0_/F ratio as a function of G concentration for a constant TA concentration. [Riboflavin] = 0.6 mg L^−1^, λ_ex_/λ_em_ = 455/520 nm, pH = 7.1.

The change in F_0_/F ratio versus G concentration for both variable and constant TA concentrations were plotted in Figure 6 and Figure 7. For a constant TA concentration, the observed changes can be only attributed to the variation of G concentration. In Figure 10, the variation of the F_0_/F ratio with G concentration for all the dispersions, when TA concentration is kept constant, can be observed.

Table 6 lists the fluorescence quenching constants of riboflavin for G dispersions in TA 2.0 and 0.5 g L^−1^ for a constant TA concentration. The intercept and slope values for the linear fits along with their standard deviation (SD) and the correlation constant are also included. In these cases, K values correspond only to the presence of G because TA decreases the fluorescence of riboflavin, but in the same way in all solutions.

The multiple linear regression is also included in the table, although TA concentration has only two values (0.5 y 2.0 g L^−1^), and therefore, the influence of this variable is not significant (*p*-value = 0.54).

The F_0_/F ratio shows a linear fit with a good correlation coefficient (90.5%), nonetheless five data had to be removed, since the variability was considerably higher than the one found when plotted versus TA, especially for the dispersion with 4.0% G.

The values found for the quenching constants are higher for the lowest G percentage of each TA concentration studied, similar to the behaviour described above, for variable TA concentrations. For the dispersion with 2.0 g L^−1^ of TA and 0.5 wt% G, a K value of 0.06 was obtained when the two points corresponding to the lowest concentrations were removed (open circles in Figure 10), albeit the Stern–Volmer plot is not linear, hence data corresponding to this dispersion are highlighted in colour. For the dispersion with 1.0 wt% G, a K value of 0.029 was obtained, and for those with 2.0 and 4.0% G in 0.5 g L^−1^ TA, the values were 0.042 and 0.024 L mg^−1^ respectively. However, in these dispersions, and especially for that with 4.0%G, a high data variability was found that was repeated when different series of dispersions were prepared under the same conditions. Therefore, the relationship between the experimental data and the Stern–Volmer equation is not as good as required, and some values must be removed.

The fit obtained for the multiple regression taking, into account all the G and TA concentrations studied in this work, is worse than that calculated for variable TA concentration, and 5 data had to be removed to obtain a good correlation coefficient (90.5%). Figure S4 shows the relationship between the experimental F_0_/F values and those predicted by the mathematical model.

The different variabilities in the measurements found for the two series studied in this work may be due to the fact that when the initial dispersion is diluted with water, the G/TA ratio remains constant, hence the system formed between both compounds is stationary. However, by diluting the dispersion with tannic acid, this G/TA ratio no longer remains constant and therefore the G/AT system is being altered. In such cases, not only does the TA present in the dispersion interact with riboflavin, but also, the free TA used for the dilution interacts with it, destabilizing the system and thus altering the quenching process, causing a high variability in the data. The quenching constants obtained for variable G concentration are much lower than those obtained with variable TA concentration, which corroborates the strong influence of TA on the value of such constants due to its stronger interaction with riboflavin than G.

### 2.6. Morphology of G Dispersions in TA

**Figure 11:**
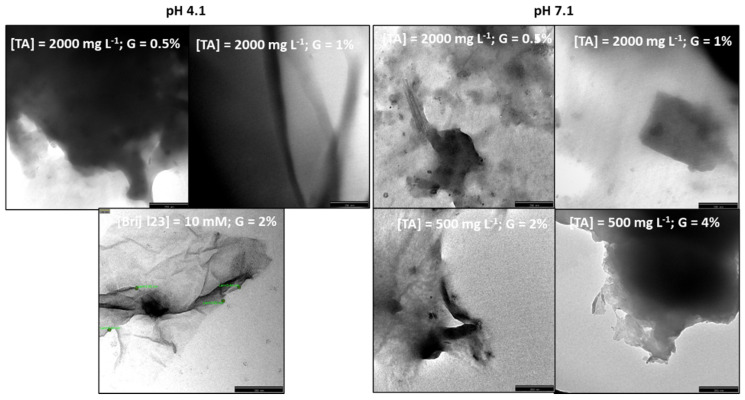
Representative TEM micrographs of G dispersions (0.5 wt% and 1.0 wt%) in TA 2.0 g L^−1^ and dispersions (2.0 wt% and 4.0 wt%) in TA 0.5 g L^−1^ at pHs of 4.1 and 7.1. 40,000× magnification. For comparative purposes, a G dispersion (2.0 wt%) in 10 mM Brij L23 is also included.

The structure and morphology of G dispersions in TA at pHs of 4.1 and 7.1 have been investigated by Transmission Electron Microscopy (TEM), and typical micrographs obtained for both pHs at different G concentrations are compared in Figure 11.

For the two pHs, the G sheets seem to be highly covered by TA, especially at low G percentages, and display a soapy aspect. Further, small black dots can be observed in the images with low G content, which could arise from TA aggregates, as reported previously for TA coatings on polymeric nanoparticles [36].

At pH 4.1, the average thicknesses obtained are 6.32 ± 0.7, 4.93 ± 0.3 and 3.58 ± 0.2 for G weight percentages of 0.5, 1.0 and 2.0 wt%, respectively. At pH 7.1, the mean values are 4.39 ± 0.6, 2.67 ± 0.5, 2.25 ± 0.4 and 2.14 ± 0.4 for G weight percentages of 0.5, 1.0, 2.0 and 4.0 wt%. Accordingly, at pH 7.1, the G sheets appear to be better dispersed and are thinner than at pH 4.1. The improved exfoliation found at a pH of 7.1 is likely related to the higher dissociation degree of the hydroxyl groups of TA. Thus, at the higher pH, TA should be negatively charged, and when adsorbed onto the G sheets, it would provoke a repulsion between the sheets. G layers wrapped in TA are stabilized against re-aggregation by the repulsive electrostatic interactions between nearby TA-coated flakes, producing quenching on the riboflavin fluorescence.

For both pHs, the thickness decreases with the increasing G percentage. This can be rationalized considering that the higher the G concentration, the lower the amount of TA in the dispersion, hence the nanomaterial is less covered by TA, and the thickness diminishes. These observations are in agreement with the results obtained from fluorescence measurements (Figure 4).

In order to compare with the morphology obtained upon dispersion of G in synthetic surfactants, a TEM micrograph of G 2.0 wt% dispersed in 10 mM Brij L23 is also included in Figure 11. In this case, the mean thickness found is 3.30 ± 0.4, comparable to those found for dispersions in TA. Indeed, the exfoliation attained herein may be as effective as that obtained for G dispersions in synthetic surfactant solutions [16], which led to flake thicknesses in the range of 1–8 nm. Further, it was found that the exfoliation level depends on the surfactant nature: the thinnest layers were observed for dispersions in the non-ionic surfactant, in which the stabilization mechanism seems to be based on steric and polar effects. Overall, TEM images obtained in this work confirm the good exfoliation of G upon ultrasonication in TA aqueous solutions and indicate that the degree of exfoliation depends on the G/TA weight ratio.

### 2.7. Comparison of the Quenching effect of TA, Synthetic Surfactants and Polymers on the Fluorescence of Riboflavin

Ionic surfactants induce fluorescence quenching on riboflavin, the highest effect caused by the anionic surfactant SDS [16]. Both cationic surfactants CTAB and DTAB attenuate the fluorescence to a lesser extent than SDS, indicating that the quenching magnitude does not depend on the length of the hydrocarbon chain, which is albeit influenced by the charge (i.e., between 0.03 and 0.09 L mg^−1^, depending on the percentage of G with respect to the surfactant). The quenching constants obtained are lower than those obtained with TA. The nonionic Brij L23 interacts strongly with riboflavin and disperses G very well, giving constants of 0.05 and 0.01 L mg^−1^ for surfactant concentrations of 0.002 and 0.010 M, respectively.

Other dispersants with a polymeric nature, such as PEG [18], provide even lower constants and the relationship between F_0_/F and G concentration is not linear like Poloxamer 407 [19]. Therefore, it can be concluded that TA is an effective dispersing agent for G, as already observed in relation to graphite exfoliation [24], and its dispersion efficiency is better at pH 7.1 than at 4.1, since the interaction of G with riboflavin is weak at this low pH. However, at pH 7.1 the exfoliation is better given that TA is negatively charged, causing the repulsion between neighbouring G coated sheets. Overall, results corroborate that this antioxidant biocompatible compound is an effective dispersant for graphene-based nanomaterials and can be used as a green alternative to conventional surfactants and synthetic polymers for the determination of biomolecules.

## 3. Materials and Methods

### 3.1. Reagents

All the reagents were of analytical grade. Graphene, made up of less than 6 sheets of thickness less than 2 nm, was supplied by Avanzare Innovación Tecnológica SL. (Logroño, Spain). Tannic acid and riboflavin were purchased from Sigma Aldrich (Madrid, Spain). Sodium hydroxide was from Panreac (Barcelona, Spain), phosphoric acid was from Sigma Aldrich (Madrid, Spain), and the ultra-pure water was obtained in a Milli-Q system from Millipore (Milford, CT, USA).

### 3.2. Instrumentation

Fluorescence spectra were recorded on a Perkin-Elmer LS-50B fluorimeter (Walthman, MA, USA) at 25 ± 1 °C equipped with a Thermomix BU bath from Braun. The excitation and emission slits were set at 5 nm. The cuvettes were quartz with 1 cm light path. The software used to record the spectra was FL WinLab from Perkin-Elmer (Walthman, MA, USA).

G dispersions were prepared with an Elmasonic S40 ultrasound bath, a Hielscher UP400S ultrasound probe (Teltow, Germany), and an Orto Alresa Digicen centrifuge (Madrid, Spain).

Transmission electron microscopy (TEM) measurements were performed on a Zeiss EM-10C/CR microscope (Oberkochen, Germany) with a voltage of 60 kV.

### 3.3. Procedure

#### 3.3.1. Preparation of the Solutions

Tannic acid solutions of 0.5 g L^−1^ and 2.0 g L^−1^ in ultrapure water were prepared at two different pHs, 4.1 and 7.1, at which some tannic acid hydroxyl groups are ionized. The pH was adjusted with a 0.01 M sodium hydroxide (NaOH) solution. A stock solution of riboflavin was prepared with 28% *v*/*v* H_3_PO_4_ buffer and stored at 4 °C in glass beakers in the dark. Working solutions were prepared by dilution in the buffer, and the riboflavin concentration for studying the interaction with TA and with G dispersions in TA was of 0.6 mg L^−1^.

#### 3.3.2. Preparation of G Dispersions in TA

Dispersions of G in TA were prepared by weighing the appropriate amount of G and adding the necessary volume of a TA aqueous solution, 0.5 g L^−1^ or 2.0 g L^−1^, until obtaining the desired G/TA mass ratio, in a final volume of 100 mL. The mixture was then placed in an ultrasonic bath for 30 min followed by sonication with the ultrasonic probe for 5 min at a power of 160 W, and then centrifuged for 1 h at 4000 rpm. The supernatant was collected, and the solid remaining at the bottom of the tube was separated.

From the supernatant, the final dispersions were prepared in two ways. First, by diluting with ultrapure water in order to obtain variable concentrations of G and TA while maintaining the G/TA weight ratio constant. The other way was performed via dilution with TA of the same concentration used to prepare the dispersion, in order to vary the G concentration while keeping the TA concentration constant, that is, the G/TA weight ratio variable. For each type of dispersion, four G weight ratios were prepared: 0.5, 1.0, 2.0, and 4.0 wt%.

#### 3.3.3. Riboflavin Fluorescence Spectra

Firstly, the fluorescence spectra of riboflavin, 0.6 mg L^−1^, were recorded, at T = 25.0 *±* 0.1 °C, as three-dimensional contour graphs to choose the optimal excitation and emission wavelengths. The recorded spectra were obtained in water, in the 0.5 and 2.0 g L^−1^ TA aqueous solutions, at the pHs of 4.1 and 7.1, and in presence of different G/TA dispersions. The initial excitation wavelength was set at 220 nm, and 25 spectra were registered with an increment of 10 nm.

Statistical calculations were performed using the Statgraphics Centurion XVII program.

#### 3.3.4. Transmission Electron Microscopy (TEM)

G dispersions in TA solutions at pHs of 4.1 and 7.1 were observed with a transmission electron microscope in order to assess the influence of the G/TA weight ratio and TA concentration on the state of dispersion of the nanomaterial. At least 20 measurements at different locations of the sample surface were carried out, and the average thickness along with the standard deviation are provided.

## 4. Conclusions

The effectiveness of TA as a dispersing agent for G in aqueous solutions has been carefully examined under different experimental conditions. TA provoked quenching of riboflavin fluorescence, and its magnitude depended on a number of parameters, including the TA concentration, the solution pH, and the G/TA weight ratio in the dispersion. Results indicate similar quenching effects for solution with pHs of 4.1 and 7.1, while it became stronger with increasing TA concentration. The interaction between both molecules was a result of hydrogen bonds between their hydroxyl groups and π-π stacking between their aromatic rings.

At pH 4.1, the fluorescence intensity was about the same in the presence and the absence of G, indicating that the nanomaterial dispersed in TA hardly alters the fluorescence of riboflavin, since the interaction between both molecules should be very weak. The sonication time applied during the preparation of the dispersions at pH 4.1 did not change the interaction of riboflavin with G.

At pH 7.1, G dispersed in TA interacted with riboflavin, hence a synergistic effect of both on attenuating the fluorescence of the vitamin was detected. The decrease in fluorescence was stronger for dispersions with the lowest G percentages given that their TA concentration is higher, and this compound is the mayor contributor to the quenching effect. For dispersions with a constant TA concentration, the quenching magnitude depends on both the G/TA weight ratio and the TA concentration. In these measurements, a high data variability was found, due to the distribution of riboflavin between the G dispersed in TA at a constant G/TA weight ratio in equilibrium and the alteration produced by the added TA with the same concentration that changes the G/TA ratio, which is difficult to attain an equilibrium position.

The quenching of riboflavin caused by TA follows the Stern–Volmer relationship up to concentrations of at least 2.0 g L^−1^ (1.2 mM), and up to G contents of 20 mg L^−1^. This linear relationship between F_0_/F and TA concentration can be used to determine the concentration of this antioxidant compound in the absence of other molecules that induce fluorescence quenching. Overall, it is demonstrated that this biocompatible molecule is an effective and environmentally friendly substitute for synthetic surfactants and polymers as dispersant for graphene-based nanomaterials and would aid in suppressing agglomerates and improving material processability and properties.

## Figures and Tables

**Table 1:** Ratio of the fluorescence intensity of riboflavin in water (F_W_) and in tannic acid 0.5 and 2.0 g L^−1^ (F) at pHs of 4.1 and 7.1 for a λ_exc_ = 455 nm and a λ_em_ = 520 nm.

pH	F/F_W_	
	[TA] = 0.5 g L^−1^	[TA] = 2.0 g L^−1^
4.1	0.67	0.39
7.1	0.74	0.36

**Table 2:** Quenching constants (K) for riboflavin in TA solutions 2.0 g L^−1^ at pHs of 4.1 and 7.1.

	Intercept ± SD	Slope ± SD	r	K, L mg^−1^
pH 4.1	1.05 ± 0.02	0.00075 ± 0.00002	0.9977	0.00075
pH 7.1	0.94 ± 0.06	0.00072 ± 0.00004	0.9920	0.00072

**Table 3:** Quenching constants (K) of riboflavin fluorescence by G dispersions (0.5% and 1.0 wt%) in TA 2.0 g L^−1^ at pH of 4.1 with constant G/AT ratio. Values at the top correspond to TA as quencher, and at the bottom, to G as quencher.

%G	a	b (TA)	r	K, L mg^−1^
0	1.05 ± 0.02	0.00075 ± 0.00002	0.9977	0.00075
0.5	0.96 ± 0.04	0.00090 ± 0.00003	0.9970	0.00090
1.0	0.99 ± 0.01	0.00066 ± 0.00008	0.9998	0.00066
		**b (G)**		
0.5	0.96 ± 0.04	0.179 ± 0.007	0.9970	0.179
1.0	0.99 ± 0.01	0.0662 ± 0.0008	0.9998	0.0662

**Table 4:** Quenching constants of riboflavin for G dispersions (0.5 wt% and 1.0 wt%) in TA 2.0 g L^−1^ at pH = 7.1 as well as G dispersions (2.0% and 4.0% *w*/*w*) in TA 0.5 g L^−1^ for a constant G/TA ratio.

**%G**	**a ± SD**	**b ± SD (TA)**	**r**	**K, L mg^−1^**
0	1.02 ± 0.02	0.00070 ± 0.00002	0.9982	0.00070
0.5	1.00 ± 0.02	0.00135 ± 0.00002	0.9995	0.00135
1.0	0.89 ± 0.08	0.00154 ± 0.00006	0.9976	0.00154
		**b ± SD (G)**		
0.5 (*n* = 4)	0.97 ± 0.02	0.2705 ± 0.005	0.9995	0.2705
1.0 (*n* = 5)	0.8925 ± 0.072	0.154 ± 0.006	0.9976	0.154
**%G**	**a ± SD**	**b ± SD (TA)**	**r**	**K, L mg^−1^**
0	1.02 ± 0.02	0.00070 ± 0.00002	0.9982	0.00070
2.0	1.00 ± 0.02	0.00199 ± 0.00008	0.9975	0.00199
4.0	1.05 ± 0.03	0.0020 ± 0.0001	0.9953	0.0020
		**b ± SD (G)**		
2.0	1.00 ± 0.02	0.100 ± 0.004	0.9975	0.100
4.0	1.05 ± 0.03	0.051 ± 0.003	0.9953	0.051

**Table 5:** Multiple linear regression analysis (95% confidence) for the fluorescence of riboflavin in the presence of G dispersions in TA for a constant G/TA ratio (*n* = 32; G wt% = 0, 0.5, 1.0, 2.0, 4.0).

F/F_0_ = a + b [G] + c [TA]					
**a ± C.I**	**b ± C.I.**	**c ± C.I.**	**R^2^, %**	**K (G), L mg^−1^**	**K (TA), L mg^−1^**
0.95 ± 0.05	0.050 ± 0.005	0.00091 ± 0.00005	95.21	0.05	0.0009

CI: 95.0% confidence interval for the coefficients; n: sample size.

**Table 6:** Quenching constants of riboflavin for G dispersions 0.5 wt% and 1.0 wt% in TA 2.0 g L^−1^ and G dispersions 2.0 wt% and 4.0 wt% in TA 0.5 g L^−1^ measured for a constant TA concentration at pH 7.1. Multiple linear regression analysis (95% confidence) is also included (**F/F_0_ = a + b [G])**.

%G; [TA]	a ± CI	b ± CI	r	K, L mg^−1^
0.5; 2.0 g L^−1^	1.01 ± 0.03	0.060 ± 0.004	0.9953	0.06
1.0; 2.0 g L^−1^	1.01 ± 0.02	0.029 ± 0.001	0.9967	0.03
2.0; 0.5 g L^−1^	1.01 ± 0.01	0.042 ± 0.002	0.9965	0.04
4.0; 0.5 g L^−1^	1.01 ± 0.05	0.024 ± 0.004	0.9683	0.02
Multiple Linear Regression (*n* = 19)	1.02 ± 0.04	0.031 ± 0.005	90.5%	0.03

CI: 95.0% confidence interval for the coefficients; n: sample size.

## Data Availability

The authors confirm that the data supporting the findings of this study are available within the article and/or its supplementary materials.

## Supplementary Materials

The following are available online at https://www.mdpi.com/article/10.3390/ijms22105270/s1, Figure S1: Representative photographs of a G 0.5% dispersion in TA 2.0 g L^−1^ at pH 4.1 (a) and pH 7.1 (b) after one week. Figure S2: Comparison of the effect of time sonication (5 and 15 min) for G 0.5 wt% dispersions in tannic acid 2.0 g L^−1^ for the two series studied in this work (λ_ex_/λ_em_= 455/520 nm; pH = 4.1. Figure S3: Multiple linear regression for F_0_/F obtained with all concentrations of G and TA in solutions with variable TA concentration. [Riboflavin] = 0.6 mg L^−1^, λ_exc_/λ_em_= 455/520 nm, pH = 7.1. Figure S4: Multiple linear regression for F_0_/F obtained with all G concentrations and TA 2.0 g L^−1^ and 0.5 g L^−1^ for a constant TA concentration 0.5 mg L^−1^ and 2.0 mg L^−1^. [Riboflavin] = 0.6 mg L^−1^ 455/520 nm, pH = 7.1.
