# Supplementary material for: Fluorescence Study of Riboflavin Interactions with Graphene Dispersed in Bioactive Tannic Acid
Source: Int J Mol Sci. 2021 May 17;22(10):5270. doi: 10.3390/ijms22105270 (PMC8156842; doi:10.3390/ijms22105270)
Supplement: Supplementary file 1 [file ijms-22-05270-s001.zip › ijms-1202108-supplementary.pdf]

SUPLEMENTARY MATERIAL

FLUORESCENCE STUDY OF RIBOFLAVIN INTERACTIONS WITH GRAPHENE DISPERSED IN BIOACTIVE TANNIC ACID

María Paz San Andrés[1,2]*, Marina Baños-Cabrera[1], Lucía Gutiérrez-Fernández, Ana María Díez-Pascual[1,2], Soledad Vera-López[1,2].

[1]Universidad de Alcalá, Facultad de Ciencias, Departamento de Química Analítica, Química Física e Ingeniería Química, Ctra. Madrid-Barcelona Km. 33.6, 28805 Alcalá de Henares, Madrid, España (Spain).

[2]Universidad de Alcalá, Instituto de Investigación Química Andrés M. del Río (IQAR), Ctra. Madrid-Barcelona Km. 33.6, 28805 Alcalá de Henares, Madrid, España (Spain).

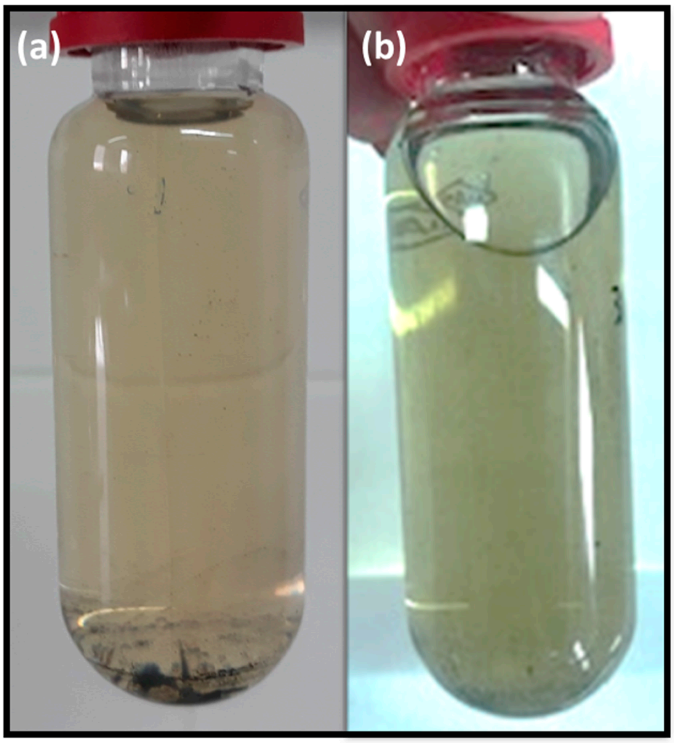

Figure S1. Representative photographs of a G 0.5% dispersion in TA 2.0 g L^{-1} at pH 4.1 (a) and pH 7.1 (b) after one week.

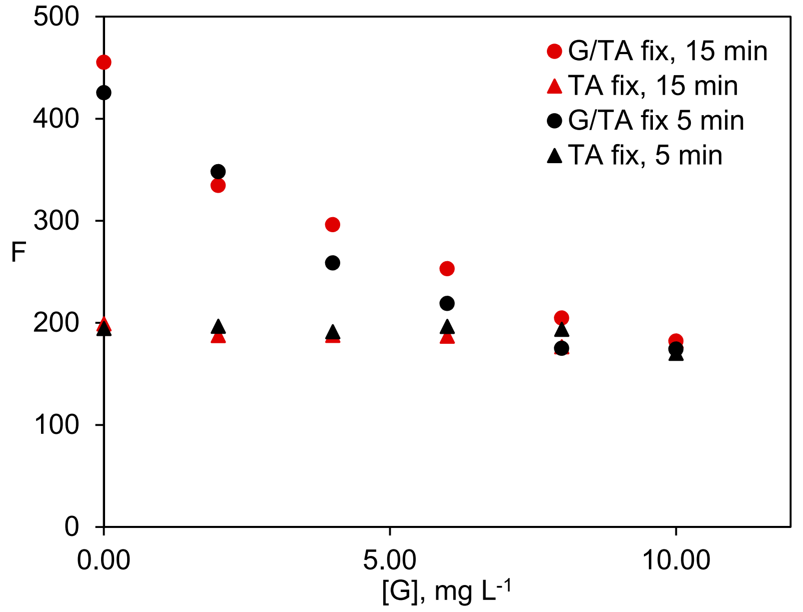

Figure S2. Comparison of the effect of time sonication (5 and 15 min) for G 0.5% w/w dispersions in tannic acid 2.0 g L^{-1} for the two series studied in this work ($\lambda_{ex}/\lambda_{em}$= 455/520 nm; pH=4.1).

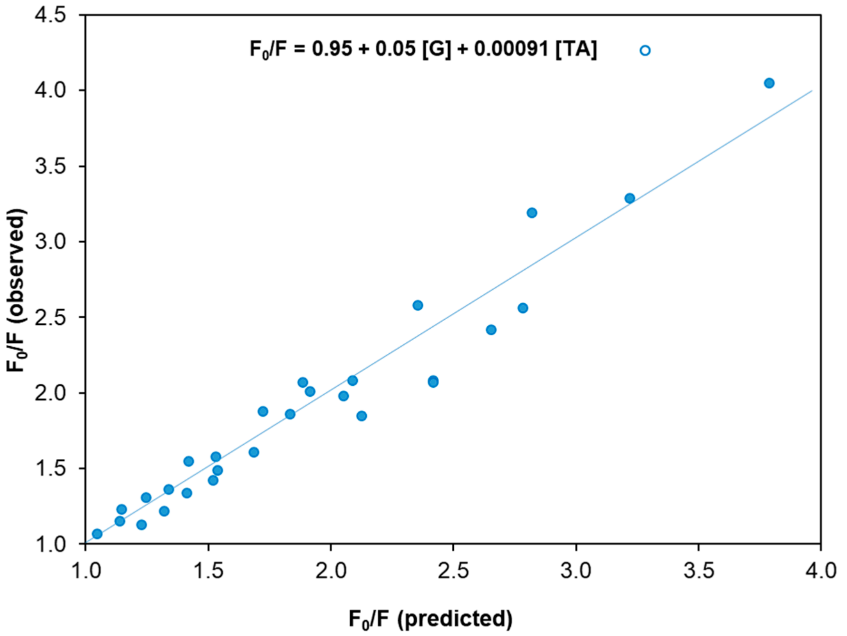

Figure S3. Multiple linear regression for F_0/F obtained with all concentrations of G and TA in solutions with variable TA concentration. [Riboflavin]=0.6 mg L^{-1}, $\lambda_{exc}/\lambda_{em}$= 455/520 nm, pH=7.1.

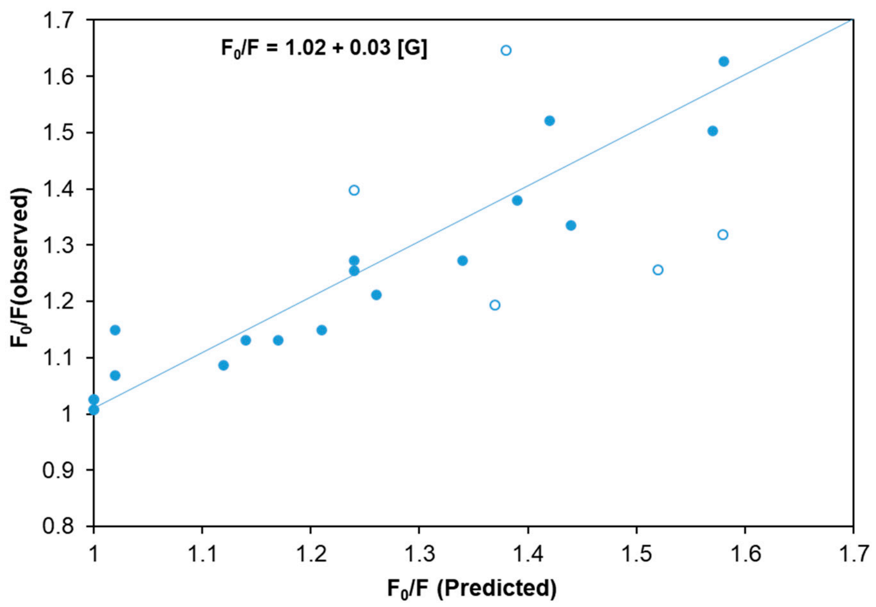

Figure S4. Multiple linear regression for F_0/F obtained with all G concentrations and TA 2.0 g L^{-1} and 0.5 g L^{-1} for a constant TA concentration 0.5 mg L^{-1} and 2.0 mg L^{-1}. [Riboflavin]=0.6 mg L^{-1} 455/520 nm, pH=7.1.
